# Influence of bacterial organic selenium on blood parameters, immune response, selenium retention and intestinal morphology of broiler chickens

**DOI:** 10.1186/s12917-020-02587-x

**Published:** 2020-09-29

**Authors:** A. M. Dalia, T. C. Loh, A. Q. Sazili, A. A. Samsudin

**Affiliations:** 1 Department of Animal Science, Faculty of Agriculture, Universiti Putra Malaysia, 43400 Serdang, Selangor Malaysia; 2 Department of Animal Nutrition, Faculty of Animal Production, University of Khartoum, Khartoum, Sudan

**Keywords:** bacterial Selenium, retention, immunity, hematology, gut morphology, broiler

## Abstract

**Background:**

Several studies indicated that dietary organic selenium (Se) usually absorbed better than an inorganic source, with high retention and bioavailability. Dietary Se as an antioxidant element affects the immune system and hematological status in animals. Therefore, the aim of this study was to evaluate the effect of dietary supplementation of bacterial selenium as an organic source on hematology, immunity response, selenium retention, and gut morphology in broiler chickens.

**Results:**

The present results revealed that supplementation of inorganic Se was associated with the lowest level of RBC, HB, and PCV with significant difference than ADS18-Se. In the starter stage, both T2 and T5 were associated with the significantly highest IgG level compared to the basal diet, while all supplemented groups showed higher IgM levels compared to the control group. In the finisher phase, all Se supplemented groups showed significant (*P* ˂ 0.05) increases in IgG, IgA, and IgM levels compared to T1. Birds fed bacterial-Se showed high intestinal villus height and better Se retention more than sodium selenite. The organic selenium of ADS18 had a superior action in improving Se retention compared to ADS1 and ADS2 bacterial Se.

**Conclusions:**

Bacterial organic Se had a beneficial effect on the villus height of small intestine led to high Se absorption and retention. Thus, it caused a better effect of Se on hematological parameters and immunity response.

## Background

Selenium (Se) is a structural component of at least 25 selenoproteins that contribute in a regulation of various biological functions in the body. Recent studies are converted from focusing on Se toxicity to its essential nutritional effects. A huge number of studies were carried out to evaluate the effects of Se on poultry nutrition and biological functions, and rapid discoveries were made to reveal the proper source of Se and the optimal level that must be added to the poultry diet. Selenium absorption and retention in the body depend mainly on the ingested form [1]. All recent studies demonstrated that organic Se is more bioavailable than inorganic forms in poultry nutrition [2, 3]. Organic Se is usually associated with higher absorption and retention compared to the inorganic form [4]. Moreover, organic Se is retained in the muscle tissues more than inorganic Se due to their different absorption pathways. Organic Se retained in a higher level in spleen, duodenum and ileum, indicating higher Se absorption, while lower Se concentrations were found in the brain, liver, and breast [5]. Organic Se of the yeast origin showed greater Se levels in liver and breast tissues compared to birds fed elemental Se, sodium selenite and the basal diet [6]. In the study of Zhao et al., [7] organic selenium compound called 2-hydroxy-4-methylselenobutanoic acid (SeO) showed a unique ability to enrich selenomethionine and total selenium depositions more than sodium selenite and selenium yeast. Which induce the early expression of some selenoproteins and to enhance the protein production of GPX4 in the tissues of chicks. According to Rajashree, et al. [8], organic Se at the level of 0.5 ppm showed high Se retention compared to inorganic form in broiler chickens.

Improvement of Se retention usually associated with better selenoprotein synthesis and efficient biological functions such as immunity response and blood formation. Selenium supplementation in different sources significantly improved white blood cells (WBCs) and red blood cells (RBCs) in broiler chickens compared to sodium selenite. However among the different Se sources, elemental Se had the better effect on the immunity response [9]. Moreover, it has been reported that Se deficient diets lead to a cellular and humoral immunity damages. The benefits of selenium supplementation are to boost selenoprotein expression and immunity response and offer a more precise approach for moderating chronic inflammation [10]. Nutritional supplementation of organic selenium plays a vital role in the activity of multiple components of the animal’s immune system [11].

Recently Tong et al. [12], revealed that selenium-enriched yeast can reduce induced intestinal injury in broiler chickens with significant increases in the body weight, feed conversion ratio, villi height, and villus/crypt ratio. But rare studies have been conducted to compare the effect of organic and inorganic selenium on intestinal morphology and selenium retention. Therefore, the current study sought to examine the impact of various organic bacterial selenium sources compared to the inorganic form on hematology, immunity response, selenium retention, and gut morphology in broiler chickens.

## Results

### Hematological parameters

The effects of inorganic and bacterial organic Se on the whole blood parameters of 42-day-old broilers are shown in Table 1. There was significant reduction of RBC, Hb and PCV in birds supplemented with inorganic Se (T2), whereas an increase in organic fed group particularly ADS18. However, the distinct was between inorganic (T2) and organic (T5) Se supplemented birds. Moreover, T2, T3, and T5 showed significant reduction (*P* < 0.05) in WBC compared to the basal diet, while, no significant difference was observed between T1 and T4. The remaining hematological parameters were unaffected by the treatment’s effects.

**Table 1:** Effect of inorganic and bacterial organic selenium on serum hematological parameters in broiler chickens

Dietary Treatments^1^								
**Parameters**	**T1**	**T2**	**T3**	**T4**	**T5**	**SEM**	***P***	**Ref**
RBC × 10^12^/L	2.47 ^ab^	2.25 ^b^	2.47 ^ab^	2.47 ^ab^	2.64 ^a^	0.05	0.037	1.82–3.46
HB g/L	114.3 ^ab^	106.6 ^b^	117.5 ^ab^	118.0 ^ab^	125.5 ^a^	2.1	0.011	79–159
PCV L/L	0.277 ^ab^	0.258 ^b^	0.282 ^ab^	0.280 ^ab^	0.300 ^a^	0.01	0.025	0.25-048
MCV Fl	112.8	115.0	114.0	113.3	113.5	0.75	0.912	100–200
MCHC g/L	411.5	413.5	416.3	421.7	419.0	2.68	0.820	376–456
WBC × 10 ^9^/L	39.5 ^a^	28.2 ^bc^	21.4 ^c^	37.3 ^ab^	18.7 ^c^	2.73	0.006	13.84–37.82
Hetro × 10 ^9^/L	23.8	18.8	12.4	21.6	12.2	1.78	0.128	1.68–25.42
Lymp %	18.8	21.5	27.0	25.3	18.5	1.36	0.170	12.34–32.78
Mono %	8.3	6.0	7.5	7.0	5.5	0.66	0.768	2.52–12.3
Eosin %	2.8	2.5	3.0	3.7	3.0	0.28	0.797	2.06–3.89
Baso%	8.5	3.8	5.0	6.7	7.5	0.93	0.489	4.67–9.86
Thrombo × 10 ^9^/L	9.1	2.4	2.6	1.1	1.8	0.3	0.277	0.95–11.82

^1^T1; basal diet, T2; basal diet + 0.3 mg/ kg feed sodium selenite, T3; basal diet + 0.3 mg/ kg feed ADS1 Se, T4; basal diet + 0.3 mg/ kg feed ADS2 Se, T5; basal diet + 0.3 mg/ kg feed ADS18 Se

^a,b,c^Means in the same row with different superscripts are significantly different. Ref; refrence values according to Haematology & Clinical Biochemistry Laboratory, Faculty of Vetrinary Medicine. UPM

### Plasma immunoglobulins

The effects of inorganic and bacterial organic Se on plasma IgG, IgA, and IgM concentration are shown in Table 2. The dietary Se supplementation resulted to significant differences (*P* < 0.05) of IgG and IgA in the initial (starter) phase of broiler birds. Higher IgG level was observed with birds in T2 and T5, thus, IgA was significantly higher in T3 compared to control (T1) groups, respectively. Neither the inorganic nor the organic Se sources were shown to be better than the other, however they were all better than the control for IgM. A contrary trend was observed in finisher phase. There was significant (*P* < 0.05) increase in IgG, IgA, and IgM among all the Se supplemented groups compared to control, although, no significant differences exist within the Se supplemented groups either.

**Table 2:** Effects of inorganic selenium and different sources of bacterial organic selenium on plasma immunoglobulin levels in broiler chickens

Dietary Treatments^1^								
**Parameters**	**T1**		**T2**	**T3**	**T4**	**T5**	**SEM**	***P*-value**
DAY 21								
IgG (mg/mL)		133.1^b^	364.6^a^	298.5^ab^	298.7^ab^	372.0^a^	33.38	0.032
IgA (ug/mL)		763.8^b^	743.0^b^	1335.7^a^	754.8^b^	1085.2^ab^	70.78	0.022
IgM (ug/mL)		481.6^c^	552.2^ab^	502.7^b^	608.4^a^	508.5^b^	37.13	0.041
DAY 42								
IgG (mg/mL)		258.4^b^	469.0^a^	454.4^a^	450.1^a^	476.4^a^	25.66	0.045
IgA (ug/mL)		1156.9^b^	1294.5^a^	1202.4^a^	1193.4^a^	1117.6^a^	75.01	0.014
IgM (ug/mL)		690.5^b^	840.9^a^	760.1^a^	719.3^a^	709.0^a^	27.63	0.007

^1^T1; basal diet, T2; basal diet + 0.3 mg/ kg feed sodium selenite, T3; basal diet + 0.3 mg/ kg feed ADS1 Se, T4; basal diet + 0.3 mg/ kg feed ADS2 Se, T5; basal diet + 0.3 mg/ kg feed ADS 18 Se

^a,b,c^ Means in the same column with different superscripts are significantly different

### Selenium retention

Table 3 shows the Se retention in broiler chickens supplemented with inorganic Se and different sources of bacterial organic selenoprotein for 42 days. Selenium supplemented diets versus basal diet showed a significant difference in ingested and excreted Se compared to the negative control (T1). However, the percentage of Se retention showed a significant difference in the finishing stage with an insignificant effect in the starter stage when the basal diet was contrasted to Se-supplemented diets. Moreover, bacterial organic Se in broiler feed resulted in a significant (*P* < 0.05) increase of finisher ingested Se in contrast to inorganic Se (T2).

**Table 3:** Effects of inorganic and bacterial organic Se sources on serum and tissues Se concentration, and selenium retention in broiler chickens

Parameters	Dietary treatments ^a^					SEM	*P* value		
	**T1**	**T2**	**T3**	**T4**	**T5**		**Anova**	**B**	**O**
0–21 days									
Ingested Se µg/g	101.05^b^	403.91^a^	413.81^a^	406.80^a^	407.65^a^	32.82	< 0.0001	< 0.0001	0.0632
Excreted Se µg/g	45.71^d^	179.95^ab^	233.38^a^	170.57^b^	109.08^c^	17.41	< 0.0001	< 0.0001	0.4766
Retention %	54.76^b^	55.45^b^	46.08^b^	58.07^b^	73.24^a^	2.82	0.012	0.4694	0.4550
22–42 days									
Ingested Se µg/g	236.3^e^	705.6^d^	792.2^c^	819.9^b^	920.4^a^	63.95	< 0.0001	< 0.0001	< 0.0001
Excreted Se µg/g	118.33^c^	233.37^b^	232.75^b^	302.16^a^	256.00^ab^	17.62	0.001	< 0.0001	0.1841
Retention %	49.93^b^	66.93^a^	70.62^a^	63.15^a^	72.19^a^	2.39	0.002	0.0002	0.6183

B = basal diet VS Se supplemented diets, O = organic Se VS inorganic Se, *P* < 0.05 = significant differences

^a− c^Means with different letter within a row differed significantly

^a^T1; basal diet, T2; basal diet + 0.3 mg/ kg feed sodium selenite, T3; basal diet + 0.3 mg/ kg feed ADS1 Se, T4; basal diet + 0.3 mg/ kg feed ADS2 Se, T5; basal diet + 0.3 mg/ kg feed ADS18 Se

**Fig. 1:**
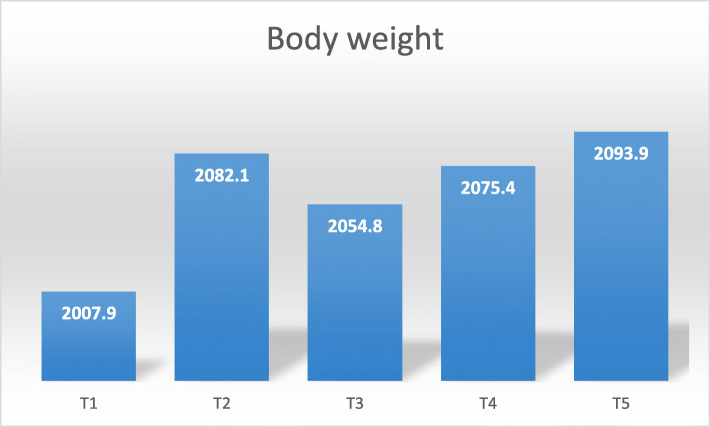
Forty-two-days body weight of broiler chicken. Treatments: T1; basal diet, T2 basal diet + 0.3 mg/kg sodium selenite, T3: basal diet + 0.3 mg/kg Se of ADS1, T4; basal diet + 0.3 mg/kg Se of ADS2, T5: basal diet + 0.3 mg/kg Se of ADS18. Bars with no common letter differ significantly (*P* < 0.05)

In the starter phase, control group was observed to have lower ingested and excreted Se. There was an insignificant difference in the ingested Se among the Se supplemented diets, however, the excreted Se was significantly (*P* < 0.05) lower in T5 compared to other dietary groups. Besides that, the retention percentage in this stage was significantly (*P* < 0.05) higher in T5 compared to other treatments. In the finisher diet, ingested Se was significantly different among treatments, which were, 236.3, 705.6, 792.2, 819.9, and 920.4 µg/g in T1, T2, T3, T4, and T5, respectively. The excreted Se was found to be higher in T4 and with lowest in T1. The Se retention percentage remain unchanged among all the Se supplemented diets and significantly (*P* < 0.05) better than the control, this could probably be connected to better body weight recorded in Se treatment groups than in the control group (Fig. 1).

### Villus height and crypt depth of the duodenum, jejunum, and ileum

The villi height and crypt depth of the duodenum, ileum, and jejunum of birds fed inorganic and bacterial organic Se after 21 and 42 days of age are shown in Table 4. At the starter stage, broilers received bacterial organic Se had significantly (P < 0.05) higher duodenum, ileum, and jejunum villi height compared to those fed the basal diet. Supplementation of bacterial organic Se showed higher jejunum villi height compared to inorganic Se (T2), also in the duodenum villi height, T4 and T5 of bacterial organic Se were significantly (P < 0.05) higher than T2. On the other hand, no significant difference was observed in the ileum villi height between the birds fed inorganic and bacterial organic Se except in T3 of bacterial organic Se, which indicated lower villi height compared to T2. Furthermore, no significant differences were observed for crypt depth in duodenum, jejunum, and ileum among all the treatments groups.

**Table 4:** Effects of inorganic and bacterial organic selenium sources on villus height and crypt depth of the duodenum, jejunum, and ileum in broiler chickens

Parameters	Dietary treatments ^1^						
	**T1**	**T2**	**T3**	**T4**	**T5**	**SEM**	***P-value***
21 days							
**Villi height µm**							
Duodenum	914.0^c^	930.9^cb^	1020.5^ab^	1109.6^a^	1141.9^a^	20.72	< 0.0001
**Jejunum**	458.23^b^	514.12^b^	599.33^a^	622.76^a^	593.07^a^	14.63	0.0002
**Ileum**	326.53^c^	484.97^a^	402.35^b^	430.14^ab^	475.16^a^	13.18	< 0.0001
**Crypt depth, µm**							
**Duodenum**	80.87	75.15	78.35	74.17	73.88	1.82	0.7199
**Jejunum**	72.07	71.63	70.75	71.83	73.21	1.51	0.9925
**Ileum**	75.36	75.59	70.80	72.69	71.82	2.21	0.9508
42 days							
**Villi height µm**							
**Duodenum**	1159.9^c^	1163.9^c^	1155.1^c^	1200.1^b^	1265.2^a^	16.71	0.0360
**Jejunum**	619.74	625.57	685.19	731.08	696.85	16.53	0.1474
**Ileum**	550.62	575.92	576.10	588.88	599.52	13.92	0.8618
**Crypt depth, µm**							
**Duodenum**	97.78	99.68	94.99	93.10	92.91	2.36	0.8809
**Jejunum**	89.68	87.14	85.33	83.20	75.99	1.51	0.0610
**Ileum**	91.61	87.75	82.81	80.99	78.81	2.38	0.4477

^1^T1; basal diet, T2; basal diet + 0.3 mg/ kg feed sodium selenite, T3; basal diet + 0.3 mg/ kg feed ADS1 Se, T4; basal diet + 0.3 mg/ kg feed ADS2 Se, T5; basal diet + 0.3 mg/ kg feed ADS18 Se

^abc^ Means with different letter within a row differed significantly

At the finisher phase, birds fed diet T4 and T5 had significantly (P < 0.05) higher villus height in the duodenum than the inorganic Se (T2) and the basal diet (T1), respectively. There were no significant differences (P > 0.05) among T1, T2, and T3 treatments for the duodenal villus height. Moreover, there were no significant differences for villi height in the ileum and jejunum among the all treatment group. The experimental diets had no effects on the duodenum, jejunum, and ileum crypt depth.

## Discussion

Hematological indices are good indicators of the animals’ physiological status and have a positive correlation with the animals’ nutritional status [13]. Selenium deficiency is associated with high generation of reactive oxygen species and exposure of erythrocytes to high degrees of oxidative stress [14]. Glutathione peroxidase is an enzyme that plays a major role in protection of erythrocytes and hemoglobin in erythrocytes against free radicals and oxidative stress. This enzyme contains selenium and therefore selenium is indirectly involved in the prevention of oxidative damage to erythrocytes [15]. Results obtained by Okunlola et al. [16], demonstrated that Se supplementation in different levels had no effect on PCV, HB, RBC and, WBC, whereas, significant differences (P < 0.05) was reported in heterophyl and lymphocytes. According to Chen et al. [3] and Boostani et al. [17], different selenium sources had no effect on blood WBC, RBC, HB, and PLT of broiler chickens. In contrast, Biswas et al. [18] and Fawzy et al. [19], reported that Se supplementation increased the erythrocytes counts in poultry and changed PCV and HB significantly, and this supports the finding of this study that, supplementation of bacterial organic Se of (T5), showed significantly higher level of RBC, HB, and PCV compared to sodium selenite. Also, the finding of the current study indicates a significant (P > 0.05) reduction in WBC in T2, T3, and T5 compared to the basal diet, with no significant differences in monocyte, eosinophil and basophil percentages. The present finding is not in agreement with Fawzy et al. [19] and Singh et al. [20] on supplementation of Se enhances cell mediated-immunity, and significantly increases WBC. In the present study, the WBC count of Se supplemented treatments was lower than that of the basal diet but still within the reference range according to Mitruka and Rawsley, [21]. In the present study, sodium selenite reduced RBC, PCV, and HB values compared to bacterial organic Se, although all levels are still in the normal range according to Schalm et al. [22] and Mitruka and Rawsley, [21]. The observed reduction of RBC, PCV, and HB due to sodium selenite supplementation, may indicate the high generation of reactive oxygen species of sodium selenite in compare to organic Se. Sodium selenite can produce free radicals which adversely affects the blood formation through denaturation of hemoglobin, and causes of hemolysis that reduced life-span of circulating erythrocytes [23]. On the other hand, in rats, dietary sodium selenite for more than one month was associated with decreases in RBC, PCV, WBC, and HB levels [24]. Also, repeated selenite treatment may reduce hemoglobin synthesis and induce a condition of hypochromic anemia [20], this may be attributed to the potential production of reactive oxygen species associated with chronic selenite supplementation. Dietary selenium supplementation improves lymphocyte divisions which is followed by a better immune response. Moreover, Se supplementation exerts its effects on immunity response mostly through its incorporation into selenoproteins such as glutathione peroxidases (GPXs), thioredoxin reductases (TXNRDs), iodothyronine deiodinases (DIOs), and selenophosphate synthetase 2 (SPS2). For non-enzymatic selenoproteins, the best characterized in terms of immune cell function is selenoprotein K (SELENOK) [10]. In the current study, both inorganic Se and bacterial organic Se of (ADS18) showed a significant increase in IgG concentration in the starter phase compared to the basal diet, but a bacterial organic Se of (ADS1) and of (ADS2) showed the highest IgA and IgM levels respectively, compared to other treatments. In the finisher stage dietary Se raised both IgG, IgA, and IgM concentrations with no significant difference between inorganic and bacterial organic Se. These data are in line with Lu et al. [25], who reported that Se-enriched exo-polysaccharides (Se-ECZ-EPS) produced by *Enterobacter cloacae* Z0206 showed a significant increase in serum antibody titers against Newcastle disease virus in birds treated with 840 mg/kg Se-ECZ-EPS. They also partially agree with the finding that supplementation of different nano- Se levels in broiler chickens had no effect on the serum IgG, IgM, and IgA of the starter phase, while the birds supplemented 0.3 mg/kg of nano-Se showed the highest IgG and IgM levels on day 42 [26]. Also, supplementation with organic and nano-Se resulted in an increasing IgM and IgG concentration compared to the other groups in oxidative and non-oxidative conditions [17]. However, some studies revealed that supplementation of different Se sources (organic and inorganic) did not affect immunoglobulins IgG, IgA, and IgM concentrations in the gilt or piglet [27]. Furthermore, Chen et al. [3], stated that both organic and inorganic Se had no effect on serum immunoglobulins at days 21 and 42. The current finding indicate the role of Se as antioxidant in preventing and maintain more developed B cell lymphocytes, which is lead to more immunoglobins productions [28]. Moreover, Se supplementation induce cytokines secretion from lymphocytes of type 2 T helper cells (Th2 cells) include Interleukin 4, 5, and 13, which are necessary for starting humoral immunity to specialize B cell lymphocytes for production of immunoglobins [29]. Therefore, dietary Se supplementation played a higher role in promoting humoral immune status in the starter and finisher stages in broiler chickens, but the effect of Se source is no longer observed. In the present study, all birds were vaccinated, which contributed to the dietary component in promoting a greater antigenic stimulation and production of a higher concentration of immunoglobulins. The fluctuation of immunoglobulin concentration observed between the two dietary stages may be attributed to the fact that the antigen that activated the immune response will decline and then most of the T cells will die, which indicates the feedback mechanism of the immune response.

Generally, organic Se may have better bioavailability and more efficient retention in the body than sodium selenite [30]. Sodium selenite is absorbed less efficiently and excreted at a higher rate compared to organic Se [31]. A study conducted by Yoon et al. [32] to examine the effect of two sources of Se-yeast as an organic Se and sodium selenite in broiler chickens, revealed that organic Se sources were more bioavailable and retained more efficiently than sodium selenite. Also, Hu et al. [33], indicated that Se retention in the whole body was higher in the group fed nano-Se compared to the group fed sodium selenite. In the current study, broilers fed dietary organic Se of T5, which originated from (ADS18) bacterial strain, retained more (P ˂ 0.05) Se in the body associated with less Se excretion than sodium selenite and other bacterial Se sources at week 3, although, at week 6, they also retained the highest Se level compared to other treatments however, the difference was insignificant. The observed difference compared to the sodium selenite may be due to the fact that sodium selenite having an ability to be bound by mucosal tissues to become unavailable for transfer to the other tissues [34]. Also, because the efficiency of Se from an organic source is related to SeMet content, it could be that organic Se of (ADS18) contains a high amount of SeMet. Therefore, it would be more interesting to investigate the type of Se in each bacterial strain, which could explain why other bacterial strains (ADS1 and ADS2) were not different from sodium selenite.

Measurement of intestinal villus height and crypt depth as morphometric characteristics are important to maintaining normal small intestine for proper absorption of nutrients and preventing translocation of bacteria from the gut [35]. The present results showed that the supplementation of bacterial organic Se had a beneficial effect on villus height in all parts of the small intestine of the starter phase, as well as in the duodenum part of the finisher phase. However, inorganic Se had no effect on the villus height compared to a basal diet except in the starter ileum part. Moreover, both inorganic and bacterial organic Se showed no effect on the crypt depth. This result is partially in line with Zamani-Moghaddam et al. [36], who indicated that supplementation of nano-Se to broiler chickens had a positive influence on villus height in all intestinal parts except the ileum, while the organic Se increased all morphometric parameters in the jejunum part. Ahmed et al. [37], reported that dietary organic Se of Se-yeast had a significant effect on duodenum and jejunum villi height in goat, but did not affect the villus height of ileum. Moreover, a study of Read-Snyder et al. [38], showed that organic Se supplementation in the form of (Sel-Plex) was associated with the greater intestinal villus height compared with the control and sodium selenite-fed birds in both normal and virus-infected groups of broiler. The main function of the small intestine is the digestion and absorption of nutrients. It is well recognised that a shortening of the villi will minimise the surface area for nutrient absorption, and a deeper crypt indicates fast tissue turnover [39]. A shortened villus height and a greater crypt depth are directly correlated with rising enterocyte turnover [40]. On the other hand, dietary antioxidants played a very important role in the enterocytes protection from apoptotic oxidative stress and could improve their development [41]. According to Tong et al. [12], Organic Se in the form of Se- yeast in broiler chickens, increased the intestinal villi height and villus/crypt ratio with significant elevation of an antioxidants and immunity response. Therefore, the improvement in the villus height in the current study may be due to the role of organic Se as an exogenous antioxidant factor, which may positively affect enterocytes viability via the active contribution of Se in intestinal glutathione peroxidase (GSH-Px2).

These findings suggest that bacterial organic Se has the potential role to improve the small intestine villus heights, especially in the duodenum segment which is the main part of Se absorption [42]. The observed differences between organic Se and sodium selenite could be due to the fact that selenite having the ability to be bound by mucosal tissues and thus become unavailable for transfer to the other tissues [34]. These observations suggest that the improved Se retention and assimilation efficiency observed in the birds fed bacterial organic Se particularly (ADS18) can be explained by improved integrity of the intestinal tract and possibly by the improved gut antioxidant status.

## Conclusions

In conclusion, the findings of the current study indicate that selenium is an essential micronutrient in improving the intestinal integrity and immunity response. The supplementation of different sources of bacterial organic Se showed high intestinal villus height and better Se retention more than sodium selenite (inorganic source). Selenium extracted from ADS18 bacterial strain had a superior action in improving Se retention compared to ADS1 and ADS2 bacterial Se. Resulted improvement in Se retention caused a significant enhancement in blood formation and serum antibodies. Supplementation of bacterial organic Se of ADS18 increased the level of the blood cells, moreover all Se sources increased the immunoglobulins levels compared to the basal diet.

## Methods

### Extraction of bacterial selenium content

fIn the current study, Se enriched bacterial strains identified as *Enterobacter cloacae* (ADS1), *Klebsiella pneumoniae* (ADS2), and *Stenotrophomonas maltophilia* (ADS18) were used as a source of bacterial organic Se. The stock culture of ADS1, ADS2, and ADS18 strains prepared at the Laboratory of Microbiology, Department of Animal Science, Faculty of Agriculture, Universiti Putra Malaysia (UPM) and the sonicated Se-enriched bacterial cells were produced according to the procedure described by Dalia et al. [43]. The extraction of selenoprotein from Se-enriched bacterial cells was carried out using dialysis technique The dialysis process was performed using dialysis sacks of flat width 25 mm, 12,000 Da, (Sigma-Aldrich) against deionised water, which was changed every 12 h for a total of 96 hours to separate inorganic Se from organic form [44]. The content in the dialysis tube was lyophilised and then used as a source of bacterial Se.

### Birds and experimental procedure

**Fig. 2:**
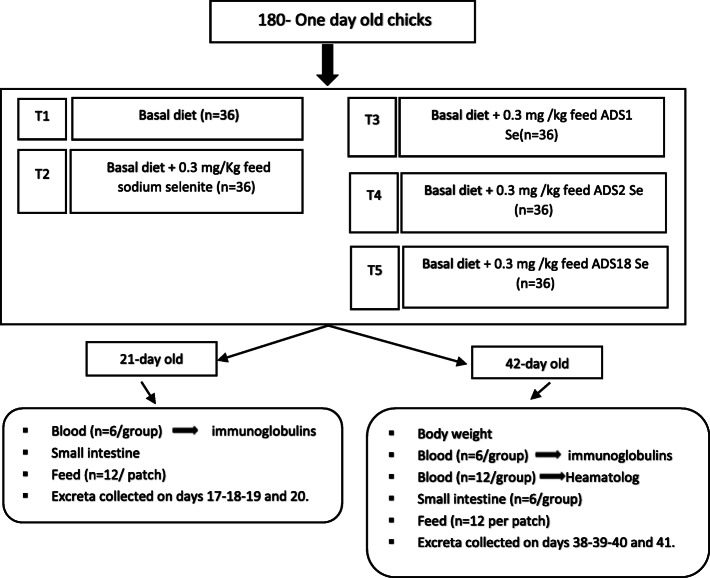
Schematic diagram of the inorganic and organic selenium feeding trial. N values present the number of chicks per feeding group or the number of taken samples

The birds handling and use in this study was carried out in compliance with the research policy guidelines of UPM on Animal Welfare and Ethics. As in (Fig. 2), a total of 180 one-day-old commercials (Cobb 500) female broiler chicks averaging 40 ± 0.13 g supplied by a local hatchery were randomly divided into five treatments fed the basal diet (Table 5), each with six replicates of 6 birds per replicate. The dietary treatments consisted of the basal diet supplemented with (0.3 mg/kg feed sodium selenite), (0.3 mg /kg feed ADS1 Se), (0.3 mg /kg feed ADS2 Se), and (0.3 mg /kg feed ADS18 Se) in addition to the basal diet treatment served as a control group. Starter diet was offered from 0 to 3 weeks old and finisher from 4 to 6 weeks old. Water and feed were given ad libitum to all the chickens. Experimental birds were housed in UPM- farm (Ladang-2) using the semi-closed system. Lightening was 12 h per day. All the birds subjected to vaccination against bronchitis (IB) and Newcastle disease (ND) on day 7, and against infectious bursal disease on day 14 through the intraocular route.

**Table 5:** Ingredients and nutrient content of the basal diet

Ingredients	Starter	Finisher
	%	%
Corn	52.5	56.250
Palm oil	5.00	6.00
Soybean meal (44% cp.)	32.50	30.00
Fish meal (58% cp.)	5.15	3.25
L-Lysine	0.25	0.25
DL-Methionine	0.25	0.25
Dicalcium phosphate 18% ^a^	1.60	1.85
Calcium carbonate	0.60	0.35
Salt	0.30	0.30
Mineral Premix^b^	0.15	0.15
Vitamin Premix^c^	0.10	0.10
Toxin Binder^d^	0.15	0.15
Choline Chloride	0.10	0.10
Wheat pollard (QL)	0.135	1.00
**Calculated nutrient content (g/kg DM)**^e^		
ME (MJ/Kg)	12.9	13.20
Crude protein	22.04	20.09
Crude fat	7.57	8.004
Calcium	1.189	1.0440
Phosphorus	0.786	0.768
Avail. P for Poultry	0.472	0.450
Analyzed Se (mg/kg)^f^	< 0.09	< 0.09

^a^di calcium phosphate provides phosphorus and calcium in a ratio of 1:1

^b^Mineral premix provided the following per kg diet: iron 120 mg, manganese 150 mg, copper 15 mg, zinc 120 mg, iodine 1.5 mg, and cobalt 0.4 mg

^c^Vitamin premix provided the following per kg diet: Vitamin A (retinyl acetate) 10.32 mg, cholecalciferol 0.250 mg, vitamin E (DL-tocopheryl acetate) 90 mg, vitamin K 6 mg, cobalamin 0.07 mg, thiamine 7 mg, riboflavin 22 mg, folic acid 3 mg, biotin 0.04 mg, pantothenic acid 35 mg, niacin 120 mg and pyridoxine 12 mg

^d^Toxin binder contains natural hydrated sodium calcium aluminium silicates

^f^The Se content measured using ICP.MS

^e^The diets were formulated using feedlive International software (Thailand)

### Hematological parameters

On completion of the experiment, twelve birds from each dietary treatment (2 birds from each replicate) were selected at random, then weighted and sacrificed. The slaughter procedure was conducted at the Department of Animal Science slaughterhouse, Faculty of Agriculture, Universiti Putra Malaysia. The animals were humanely slaughtered by a licensed slaughter man. The procedure involved severing the carotid artery, jugular vein, trachea and esophagus. Blood samples were taken directly from the neck vein into a vacuum tube (BD Vacutainer®, NJ. USA) containing an anticoagulant (EDTA) and directed to hematological analysis using hematology analyzer (CELL DYN 3700, Abbott USA).

### Plasma immunoglobulin concentration

At days 21 and 42, 6 birds per treatment were randomly selected and blood samples were collected into vacutainer tubes containing ethylene diamine tetra acetic acid (EDTA). Blood samples were mixed gently before storage in ice, followed by centrifuging at 3000 rpm for 15 min at 4 °C, and the plasma was stored at − 80 °C until antibody analysis.

Plasma IgA and IgM were determined using (Chicken IgA ELISA, Immunology Consultants Laboratory, Inc. USA) and (Chicken IgM ELISA, Immunology Consultants Laboratory, Inc., USA), while Chicken IgG determined using (CEA544Ga, Enzyme-linked Immunosorbent Assay Kit, Cloud-Clone Corp., USA). All the analysis performed according to the procedure recommended by the manufacturer. The absorbance was measured at 450 nm wavelength using a micro-plate reader (Infinite® 200 PRO, TECAN).

### Selenium retention

Twelve representative samples from each batch of feed (starter and finisher diets) were collected randomly and kept at − 20 °C until further analysis. Total excreta collection was performed on days 17, 18, 19, and 20 for a starter diet and on days 38, 39, 40, 41 for finisher diet. Feed and fecal samples were analyzed for Se concentration using ICP.MS. Determination of Se retention was calculated using a mass balance method [45] as follows:

Se retention (%) = (Ingested Se − Excreted Se) × 100.

### Ingested Se

Ingested Se = daily feed intake × analytical feed Se concentration.

Excreted Se = daily feces weight × feces Se concentration.

### Histomorphology of small intestine

Intestinal morphology was done employing the method stated by Choe et al. [46]. The intestinal samples of 6 birds/ treatment were collected at days 21 and day 42. Approximately 5 cm segments of the ileum (midway between the Meckel‘s diverticulum and ileo-caecal junction), a middle portion of the duodenum (apex section), and jejunum (midway between the endpoint of the duodenal loop and Meckel‘s diverticulum) were cut gently and washed with phosphate buffer saline (PBS) and fixed in 10% neutral buffered formalin. Then, the intestinal samples were dried for 16 h in an automatic tissue processor (Leica ASP 3000, Tokyo, Japan) and embedded in paraffin wax following a paraffin embedding system (Leica EG 1160, Japan). Each sample was cut at 4 µm with a rotary microtome machine (Leica RM 2155, Japan). Sections of size 4 mm were fixed on glass slides, heated at 57oC until dried, and stained with hematoxylin and eosin. The distance from the tip of the villi to the villus crypt junction represented the villus height, while, crypt depth was described as the depth of the invagination between 2 villi and was determined to employ Image-Pro Plus software as described by Touchette et al. [47]. A total of 5 villi sections per slide were evaluated in each of 6 replicate slides per intestinal sample (30 measurements for each sample) and studied with a light microscope (Dialux, LeitzWetzlar, Germany) fitted with a digital camera (Laice, Germany).

### Statistical analysis

Data were analyzed using one-way analyses of variance (ANOVA) using the Proc GLM procedure of SAS software (SAS Institute Inc., Cary, NC). The assumption of normality was by using the visual assessment of histogram distribution and Quantile-Quantile (Q-Q) plots of model residual. Duncan Multiple Range Test was used for comparisons of means for each significant difference among the treatment groups at a significant level (*P <* 0.05). The F test was also performed to determine the orthogonal contrasts among treatments.

(1) Basal diet vs. Se supplemented diets,

(2) Sodium selenite vs. bacterial organic Se,

Values of *P* < 0.05 were accepted as significant.

## Data Availability

The datasets used and/or analysed during the current study are available from the corresponding author on reasonable request.

## Abbreviations

Se: Selenium
ADS1: *Enterobacter cloacae*
ADS2: *Klebsiella pneumonia*
ADS18: *Stenotrophomonas maltophilia*
RBCs: Red blood cells
HB: Hemoglobin
PCV: Packed cell volume
IgA: Immunoglobulin A
IgG: Immunoglobulin G
IgM: Immunoglobulin M
